# Aerobic Intraoperative Abdominal Cavity Culture Modifies Antibiotic Therapy and Reduces the Risk of Surgical Site Infection in Complicated Appendicitis with Peritonitis

**DOI:** 10.1007/s11605-023-05736-3

**Published:** 2023-06-20

**Authors:** Víctor Manuel Quintero-Riaza, Romario Chancí-Drago, Natalia Guzmán-Arango, Pablo Posada-Moreno, Tatiana López-Sandoval, Isabel Cristina Ramírez-Sánchez, Johanna Marcela Vanegas-Munera

**Affiliations:** 1 Hospital Pablo Tobón Uribe, Medellín, Colombia; 2 School of Health Sciences, Universidad Pontificia Bolivariana, Medellín, Colombia

**Keywords:** Complicated appendicitis, Intraoperative culture, Drug resistance, Surgical site infection, Postoperative outcomes

## Introduction

Appendectomy is one of the most performed surgeries and is the leading cause of acute abdomen.^[1]^ The usefulness of intraoperative cultures in complicated appendicitis is controversial.^[2]^ The current evidence presents important methodological limitations and studies are needed to evaluate outcomes in patients with intraoperative cultures. We analyzed the impact of intraoperative aerobic culture on the modification of antibiotic therapy and the clinical evolution of patients with complicated appendicitis.

## Methods

Retrospective cohort study of 144 adults with complicated appendicitis (localized or generalized peritonitis) in a high complexity hospital in Colombia from 2014 to 2022. The intraoperative sample was taken according to the attending surgeon’s decision. The procedure of how the cultures were taken and analyzed can be found in the supplementary material. The primary outcomes included the modification of antibiotic therapy, surgical site infection (SSI) during the first 30 postoperative days, reintervention, length of hospital stay, and mortality. All patients were evaluated in a postsurgical outpatient visit. A Cox proportional hazards model was used with the subsequent reporting of the hazard ratio. The project was approved by the Health Ethics Committee (Act-18, 2021).

## Results

**Fig. 1:**
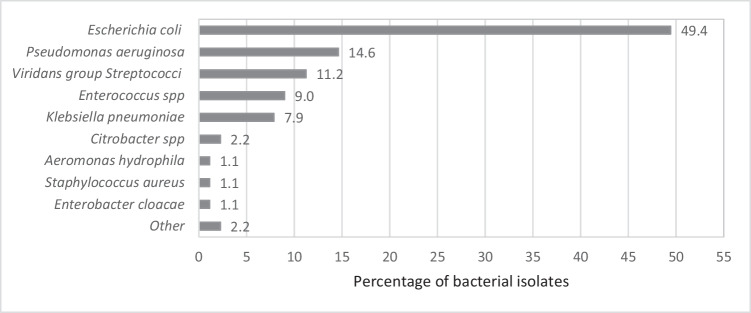
Percentage of isolated bacteria from aerobic cultures in the intraoperative period in patients with complicated appendicitis

**Fig. 2:**
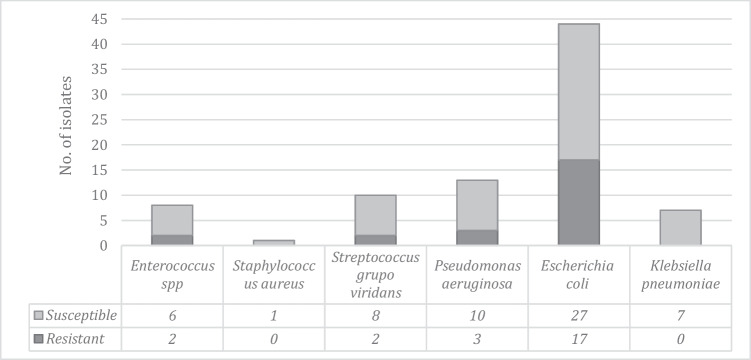
Beta-lactam-resistant bacteria in patients with complicated appendicitis. In total, 2/10 isolates of the *Streptococcus viridans* group and 2/8 isolates of *Enterococcus* spp. presented resistance to ampicillin. 1/13 isolates of *Pseudomonas aeruginosa* showed resistance to fluoroquinolones. No *Candida* spp. were isolated in any patient

Intraoperative culture was collected in 42.4% (*n*=61) patients, 56.2% had a laparoscopic approach. In most cases, the chosen empiric antibiotic as well as the therapy duration was guided according to the hospital protocol (Table 1); 86.9% of the patients had a positive culture result. The most isolated microorganisms were *Escherichia coli* and *Pseudomonas aeruginosa* (Fig. 1). *E. coli* resistance to beta-lactams was detected in 38.5% (*n*=17) of the cultures. Four of them were positive for extended-spectrum beta-lactamase (ESBL), and 13 presented inhibitor-resistant TEM. Three of the 13 isolates of *P. aeruginosa* were found to be resistant to beta-lactams, one of them was resistant to carbapenems (Fig. 2).

**Table 1:** Clinical and demographic characteristics of patients with complicated appendicitis, according to the performance of intraoperative abdominal cavity culture (Total of patients with appendicitis: 1911; complicated appendicitis *n*= 144)

Characteristics	Total *n*= 144 no. (%)	With culture *n*=61 no. (%)	Without culture *n*=83 No. (%)	*p* value
Median age (IQR)	41 (28-57.5)	44 (31-56)	38 (26-58)	0.393
Gender				0.259
Male	74 (51.4)	28 (45.9)	46 (55.4)	
Female	70 (48.6)	33 (54.1)	37 (44.6)	
Empiric antibiotic*	143 (99.3)	61 (100)	82 (98.8)	0.390
Ampicillin/sulbactam	7 (4.9)	1 (1.6)	6 (7.3)	
Cefazolin + metronidazole	90 (62.9)	29 (47.5)	61 (74.4)	
Ciprofloxacin + metronidazole	8 (5.6)	2 (3.3)	6 (7.3)	
Piperacillin/tazobactam	35 (24.5)	28 (45.9)	7 (8.5)	
Others	3 (2.1)	1 (1.6)	2 (2.4)	
Type of surgery				<0.001
Laparoscopic	81 (56.2)	32 (52.5)	49 (59.0)	
Midline laparotomy	17 (11.8)	15 (24.6)	2 (2.4)	
Rocky Davies	46 (31.9)	14 (22.9)	32 (38.5)	
Comorbidities	59 (41.0)	25 (41.0)	34 (41.0)	0.998
Diabetes mellitus	8 (5.6)	5 (8.2)	3 (3.6)	0.236
High blood pressure	22 (15.3)	12 (19.7)	10 (12.0)	0.209
Chronic kidney disease	2 (1.4)	2 (3.3)	0	0.097
Immunosuppression	2 (1.4)	1 (1.6)	1 (1.2)	0.826
Others	42 (29.2)	14 (23.0)	28 (33.7)	0.159
Home-based medicine	10 (6.9)	6 (9.8)	4 (4.8)	0.242
National Nosocomial Infection Surveillance Index (NNIS)				0.033
One	43 (29.9)	24 (39.3)	19 (22.9)	
Two	72 (50.0)	23 (37.7)	49 (59.0)	
Three	29 (20.1)	14 (22.9)	15 (18.1)	
ASA				0.830
One	17 (11.8)	7 (11.5)	10 (12.0)	
Two	86 (59.7)	37 (60.7)	49 (59.0)	
Three	38 (26.4)	15 (24.6)	23 (27.7)	
Four	3 (2.1)	2 (3.3)	1 (1.2)	

*IQR* interquartile range

^*^Institutional protocol: patients with generalized peritonitis and/or risk factors such as elderly, APACHE II score >15, poor nutritional status, presence of malignancy, colonization or infection by known multi-resistant pathogens, immunosuppression, and multiple comorbidities receive piperacillin/tazobactam for 4–7 days. Those without risk factors and without generalized peritonitis receive cefazolin plus metronidazole for 3–5 days ^[6]^

SSI was detected in 18 patients, half of these were organ-space SSI, without statistically significant difference between laparoscopic and open approaches (11.1% vs. 14.3%, *p*=0.568). The risk of presenting SSI was reduced by 73% in patients with intraoperative culture (HR: 0.27, CI95%:0.10–0.74) (Table 2, Figure 1-Supp).

**Table 2:** Association between culture performance and surgical site infection in patients with complicated appendicitis

Variable outcome	Bivariate analysis				Multivariate analysis*			
	HR	IC 95%		*p* value	HR	IC 95%		*p* value
Surgical Site Infection	0.30	0.11	0.80	0.017	0.27	0.10	0.74	0.011

^*^Adjusted by type of surgery (laparoscopy or median laparotomy or Rocky Davies) and the NNIS index. Surgical site infection is defined by the Centers for Disease Control (CDC) and the National Health Care Safety Network (NHSN) as the infectious process that appears during the first 30 days after any type of surgical procedure in cases of surgery without the application of an implant of any origin (Horan TC, et al. Am J Infect Control 2008;36:313–4)

Of the 61 patients with intraoperative culture sampling, 57.4% had a change in their antibiotic therapy once the microbiological result was obtained, resulting in a targeted antibiotic treatment in 72.1% patients. The antibiotic spectrum was reduced after the culture result in most patients (n=14/21) with initially empirical piperacillin/tazobactam treatment (Table 3). There were no statistical differences in other outcomes (Table 1-Supp).

**Table 3:** Empirical vs. targeted therapy in patients with complicated appendicitis

Empiric treatment	Targeted treatment*	Number of patients (*n*=45)
Cefazolin plus metronidazole	Ampicillin/sulbactam	1
	Cefazolin plus metronidazole	3
	Ciprofloxacin	9
	Ciprofloxacin plus metronidazole	5
	Meropenem	1
	Piperacillin/tazobactam	1
	Other	1
Piperacillin/tazobactam	Ampicillin/sulbactam	2
	Cefazolin plus metronidazole	1
	Ciprofloxacin	4
	Ciprofloxacin plus metronidazole	6
	Meropenem	1
	Piperacillin/tazobactam	7
Others	Ampicillin/sulbactam	1

^*^Targeted treatment: ampicillin/sulbactam (*n*=4; 9.1%), cefazolin plus metronidazole (*n*=4; 9.1%), ciprofloxacin (*n*=13; 29.5%), ciprofloxacin plus metronidazol (*n*=11; 25%), meropenem (*n*=2; 4.5%), piperacillin/tazobactam (*n*=8; 18.2%), other (*n*=2; 4.5%)

## Discussion

High rates of bacterial resistance in intra-abdominal infections had been reported among *E. coli* infections (14–54%), which is one of the most common isolated microorganisms.^[3]^ Colombian Association of Infectious Diseases suggests taking intraoperative cultures in patients with fibrinopurulent fluid or generalized peritonitis, to document the local epidemiology of community-acquired intra-abdominal infections and to provide target antibiotic treatment.^[4]^

Although there is evidence of positivity and resistance rates of 24% and 2% respectively for intraoperative cultures,^[2]^ 86.9% of our samples had a positive result with an overall resistance to beta-lactams of 40.6%, comparable to international reports.^[5]^ A change in antibiotic therapy was observed in 57.4% of patients, leading to targeted antibiotic therapy in 72.1% of the cases and to a reduction in antibiotic spectrum. A protective effect of intraoperative cultures on the development of surgical site infections was detected; as findings in previous studies.^[1]^ Although the low percentage of laparoscopy appendectomy mainly due to limitations in our healthcare system, there were no statistically significant differences in SSI rates between open and laparoscopic approaches.

This is a single-center study with selection biases due to non-protocolized intraoperative culture sampling.

We recommend routinely intraoperative cultures in patients with complicated acute appendicitis according to IDSA statement,^[6]^ seeking to provide targeted antibiotic management and to reduce surgical site infection, readmissions and the costs derived from hospital care.

### Supplementary Information

ESM 1

## References

[1] Son JT, Lee GC, Kim HO, Kim T, Lee D, Lee SR, Jung KU, Kim H, Chun HK (2020). Routine Intraoperative Bacterial Culture May Be Needed in Complicated Appendicitis. Ann Coloproctol..

[2] Gladman MA, Knowles CH, Gladman LJ, Payne JG (2004). Intra-operative culture in appendicitis: traditional practice challenged. Ann R Coll Surg Engl..

[3] Grupo GERMEN. Perfiles de sensibilidad a antibióticos de *Escherichia coli* aisladas en instituciones hospitalarias y laboratorios clínicos de Medellín y municipios vecinos [Internet]. Disponible en: http://www.grupogermen.org/pdf/escherichia_coli_12_14.pdf

[4] Oñate J, Gutiérrez CJP, Esparza G, Jimenez A, Medina IB, Osorio-Pinzón J (2021). Consensus Recommendations Based on Evidence for Abdominal Sepsis in the Pediatric and Adult Population of Colombia. Infectio..

[5] Antimicrobial Resistance Collaborators (2022). Global burden of bacterial antimicrobial resistance in 2019: a systematic analysis. Lancet..

[6] Solomkin JS, Mazuski JE, Bradley JS, Rodvold KA, Goldstein EJ, Baron EJ (2010). Diagnosis and management of complicated intra-abdominal infection in adults and children: guidelines by the Surgical Infection Society and the Infectious Diseases Society of America. Surg Infect (Larchmt)..

