# Supplementary material for: Aerobic Intraoperative Abdominal Cavity Culture Modifies Antibiotic Therapy and Reduces the Risk of Surgical Site Infection in Complicated Appendicitis with Peritonitis
Source: J Gastrointest Surg. 2023 Jun 20;27(11):2563–6. doi: 10.1007/s11605-023-05736-3 (PMC10661740; doi:10.1007/s11605-023-05736-3)
Supplement: Supplementary file 1 — ESM 1 [file 11605_2023_5736_MOESM1_ESM.docx]

**SUPPLEMENTARY MATERIAL**

**Culture collection and processing:**

All the samples were obtained by intraoperative aspiration with a syringe. The processing of the samples obtained from the peritoneal cavity of the patients was carried out under strict microbiological protocols. Only cultures for aerobic microorganisms were performed. The cultures consisted of differential and selective agars, as well as colony selection, identification and determination of the antibiogram using an automated method (VITEK®2). As of 2020, microbiological identification was performed by mass spectrometry (MALDI-TOF MS).

**Figure 1.** Risk of surgical site infection according to the performance of intraoperative culture in patients with complicated appendicitis

**Table 1.** Clinical outcomes according to the performance of intraoperative abdominal cavity culture in patients with complicated appendicitis

|  | **Surgical Reintervention**  **No. (%)** | **Re-entering**  **No. (%)** | **Stay Special Care Unit**  **No. (%)** | **Stay Intensive Care Unit**  **No. (%)** |
| --- | --- | --- | --- | --- |
| With intraoperative culture (n=62) | 2 (3.3) | 4 (6.6) | 1 (1.6) | 1 (1.6) |
| Without intraoperative culture (n=88) | 1 (1.2) | 7 (8.4) | 1 (1.2) | 0 |
| p value | 0.389 | 0.675 | 0.826 | 0.242 |

*There was no mortality during the study period
